# Digital volume correlation for the characterization of musculoskeletal tissues: Current challenges and future developments

**DOI:** 10.3389/fbioe.2022.1010056

**Published:** 2022-10-04

**Authors:** Enrico Dall’Ara, Gianluca Tozzi

**Affiliations:** ^1^ Department of Oncology and Metabolism, Mellanby Centre for Bone Research, University of Sheffield, Sheffield, United Kingdom; ^2^ INSIGNEO Institute for in Silico Medicine, University of Sheffield, Sheffield, United Kingdom; ^3^ School of Engineering, University of Greenwich, Chatham Maritime, United Kingdom

**Keywords:** digital volume correlation, musculoskeletal tissues, biomaterials, *in situ* mechanics, bone

## Abstract

Biological tissues are complex hierarchical materials, difficult to characterise due to the challenges associated to the separation of scale and heterogeneity of the mechanical properties at different dimensional levels.

The Digital Volume Correlation approach is the only image-based experimental approach that can accurately measure internal strain field within biological tissues under complex loading scenarios. In this minireview examples of DVC applications to study the deformation of musculoskeletal tissues at different dimensional scales are reported, highlighting the potential and challenges of this relatively new technique.

The manuscript aims at reporting the wide breath of DVC applications in the past 2 decades and discuss future perspective for this unique technique, including fast analysis, applications on soft tissues, high precision approaches, and clinical applications.

## 1 Introduction

Understanding the mechanical properties of musculoskeletal tissues and how they deform under load is fundamental to assess the effect of diseases and to optimise treatments. For example, the bone risk of fracture is associated with its local ability to resist cracking, which is associated with its local deformation (i.e., if a bone is compressed beyond 1% or stretched beyond 0.7% (Bayraktar et al., 2004) local permanent deformation and damage will occur that will lead to fracture if not healed).

However, the mechanical properties of biological tissues are complex and driven by their heterogeneous structures, which are built in spatial hierarchical arrangements. For most engineering materials, where the mechanical properties can be usually characterised evaluating only two dimensional levels (e.g., the macrostructure and the grain in metals), it is relatively easy to estimate the deformation of the structure under complex loading using homogenisation techniques. In such cases, application of contact sensors such as strain gauges are enough to measure the strain under load and simple interpolation functions can be used to accurately estimate the field of deformation. For biological tissues this is not possible. In fact, heterogeneity of the structures requires a much more detailed assessment of the local strains, hundreds or thousands of (micro)sensors would be needed to characterise the deformation of the tissue, which is not feasible nor practical. Therefore, Digital Image Correlation (DIC) has been developed to perform superficial full-field measurements of deformation (Palanca et al., 2016a). However, the complex internal structural patterns of biological tissues do not allow for simple extrapolation of the strains measured at the surface to infer internal strains. Therefore, another technique has been developed: Digital Volume Correlation (DVC, originally called “texture correlation” in (Bay, 1995) for assessing the 3D deformation of trabecular bone).

DVC requires the acquisition of 3D images of the musculoskeletal tissue in its undeformed and deformed configurations, and the application of image-processing correlation algorithms to track the heterogeneous displacement and strain fields induced by the external load. Time-lapsed three-dimensional (3D) micro-computed tomography (microCT) imaging of specimens under loading (also referred to as *in situ* mechanical testing) has enabled the study of deformation and failure mechanisms in bone and biomaterials (Bay, 1995; Nazarian et al., 2005). With DVC the displacement and strain fields can then be evaluated in 4Ds (across space and time/load level) (Roberts et al., 2014; Grassi and Isaksson, 2015).

Several DVC approaches have been developed and used to characterise the deformation in different musculoskeletal tissues, at different dimensional scales. It is clear that DVC has huge potential to unravel the mechanism of mechanical deformation within biological tissues, as highlighted by the increasing number of studies using this technique in the past 20 years: since the early 2000s more than 150 papers have been published (Search Pubmed: “Digital Volume Correlation”), of which approximately 110 are related to musculoskeletal tissues. Nevertheless, a number of challenges and potential pitfalls associated with *in situ* mechanical testing [e.g., see recent review (Dall'Ara et al., 2022)] and the application of image correlation algorithms have to be addressed in order to obtain, report and interpret DVC results in a robust way.

The goal of this mini-review is to briefly report examples of DVC applications for musculoskeletal tissues, to highlight open challenges, and to report recent developments that have the potential of further improving its applicability in musculoskeletal research.

## 2 Digital volume correlation approaches and applications

### 2.1 Local vs. global approaches

Two main families of DVC approaches (local and global) have been developed and are currently available as commercial software, freeware or customised solutions/services. In both cases probably the most important parameters for obtaining the best displacement and strain measurements with DVC are the microstructural heterogeneity of the studied tissue (i.e., distribution of features within the image domain) and the quality of the input images (i.e., signal to noise ratio).

Local approaches are based on the subdivision of the image into smaller sub-volumes and the spatial correlation of metrics computed in each of the sub-volumes of the undeformed and deformed image independently. Interpolation (e.g., tri-linear) is used to calculate displacements of voxels located between nodes. The final displacement is therefore a 3D full-field average displacement of the pattern within that sub-volume between reference and deformed volume. From the field of resultant displacement vectors for each sub-volume, the field of strain components is computed (e.g., using a centered finite difference scheme). Different parameters (i.e., correlation function, subvolume size, overlap, interpolation, etc.,) affect the precision and accuracy of the algorithm, as demonstrated for bone applications (Dall׳Ara et al., 2017). Different correlation metrics have been used, the most common being direct correlation (DC) and fast-Fourier-transform (FFT), which was found to be faster but less accurate than DC for bone applications (Palanca et al., 2016b). This approach can be quite accurate in predicting local deformations but there may be regions where the correlation is low. Therefore, it is fundamental to examine the correlation map (distribution of coefficients that provide the confidence of the local correlation) and trust the results only in the highly correlated sub-volumes. Different threshold values for local correlation have been used in the literature for different applications in musculoskeletal tissues and biomaterials. While a threshold of 60% can be considered acceptable, correlation values above 80% should be considered for optimal applications. Moreover, while for local approaches the correlation and accuracy in strain measurement is usually good within the specimen’s domain and it is acceptable close to the border, large errors are usually found outside the specimen, where little information is available. Therefore, a masking approach to remove the effect of potential noise outside the specimen is recommended (Palanca et al., 2016b). In any case, local DVC parameters should be optimised for the different applications, in function of the quality of the input images, the heterogeneity of the features visible in the images, and the level of deformation the tissue is subjected to.

Global approaches are based on the minimisation of the difference of the deformed image and the registered undeformed image when a continuous displacement field is applied. The registration equations are solved in a grid of points (e.g., regular or heterogeneous, hexahedral or tetrahedral) called nodes. Interpolation (e.g., tri-linear) within the cell of the grid is used to calculate the displacements from the nodal values. Usually a smoothing of the displacement field is applied to avoid high gradients of displacement that would lead to localised unrealistically high strains. Finally, the displacement field is differentiated into a strain field (e.g., using the finite element or finite difference methods). The global approach is usually associated to lower errors compared to the local approaches, when using the same spatial resolution (Dall׳Ara et al., 2017). This is mainly due to the choice of the regularization process through the smoothing of the displacement field during its calculation. As the object is considered a continuum, one of the advantages of the global approach is the possibility of integrating it with finite element models to account for local differences in stiffness (see Section 3.5) (Madi et al., 2013).

Local and Global DVC approaches can be also used jointly on the same given image dataset. A recent example was applied to evaluate the hydro-mechanical behaviour in natural materials, where the combined use of both approaches improved the physical interpretation of displacement and strain fields for such a complex mechanical environment (Stavropoulou et al., 2020).

Independently from the employed DVC approach, correlation parameters and in particular the sub-volume shape and size should be optimised to obtain the best spatial resolution of the approach while keeping the average (or local) error below an acceptable threshold for that specific application (Madi et al., 2013; Dall'Ara et al., 2014; Boulanaache et al., 2020). For example, the accuracy and precision of the DVC has been found to increase by increasing the size of the sub-volume, and therefore decreasing the measurement resolution following a power law (Dall׳Ara et al., 2017).

### 2.2 Applications on different musculoskeletal tissues

DVC approaches applied to CT, MRI, microCT, or Synchrotron microCT (SR-microCT; with standard absorption or phase contrast modalities) have been used to analyse the deformation patterns in different native musculoskeletal tissues at different dimensional scales. These studies have reported exhaustive measurements of the internal deformation in tissues under physiological or failure loading scenarios, enabling a much more detailed understanding of the loading and failure mechanisms of the complex hierarchical musculoskeletal tissues and their integration with biomaterials.

While reporting a comprehensive list of every study that used DVC to study the deformation in musculoskeletal tissues is not the goal of this mini-review, examples are reported in Table 1. Several organs and tissues have been studied, including for example: vertebral bodies (Hosseini et al., 2014; Hussein et al., 2018; Palanca et al., 2021), proximal femur (Ridzwan et al., 2018; Ryan et al., 2020; Martelli et al., 2021), implanted scapula (Boulanaache et al., 2020), osteochondral plugs (Tozzi et al., 2020), intervertebral disc (IVD) (Disney et al., 2019; Tavana et al., 2020a), dentin (Lu et al., 2019), bone-biomaterial (Tozzi et al., 2014; Danesi et al., 2016; Joffre et al., 2017; Pena Fernandez et al., 2019; Rapagna et al., 2019), trabecular bone (Zwahlen et al., 2015; Turunen et al., 2020; Yan et al., 2020), cortical bone (Christen et al., 2012), subchondral bone (Madi et al., 2020).

**TABLE 1:** Examples of applications of DVC at different dimensional levels to evaluate the deformation of different musculoskeletal tissues. While the goal of this minireview is not to report every study where DVC was used on musculoskeletal tissues, the reported examples provide an exhaustive overview of different applications.

References	MSK tissue	Source	N (sample size)	Dimensional scale	Imaging	Voxel size [μm]	Load	DVC algorithm	DVC spatial resolution (sub-volume size) [μm]	DVC precision strain (method) [με]	Application
Hussein et al. (2018)	Vertebral body	Human spine segment	30	Organ	MicroCT	37	Compression	Local Hussein et al. (2012)	∼4,800	630 (zero-strain)	Bone failure
Jackman et al. (2016)	Vertebral body	Human spine segment	28	Organ	MicroCT	37	Compression/Flexion	Local Hussein et al. (2012)	∼1900	NA	Bone failure and validation models
Costa et al. (2017)	Vertebral body	Porcine vertebra	4	Organ	MicroCT	∼39	Compression	Global (BoneDVC)	∼1872	∼100 (zero-strain)	Bone failure and validation models
Palanca et al. (2021)	Vertebral body with lesions	Porcine spine units	5	Organ	MicroCT	39	Compression	Global (BoneDVC)	∼1950 (several reported)	∼337 (zero-strain)	Bone failure
Danesi et al. (2016)	Vertebral body, bone cement	Porcine vertebra	8	Organ	MicroCT	38.8	Compression	Local (Davis)	∼1862	NA	Failure bone/cement interface
Palanca et al. (2016b)	Vertebral bodies w/o bone cement	Porcine vertebrae	10	Organ	MicroCT	39	Zero-strain	Global (BoneDVC) Local (Davis)	∼1872 (several reported)	Global: ∼30–40 Local: ∼60–70	Precision DVC
Tavana et al. (2020a)	IVD	Human spine unit	8	Organ	MRI (9.4 T)	90	Compression	Local (Davis)	5,040	636 (zero-strain)	Deformation IVD
Boulanaache et al. (2020)	Human scapula	Human scapula	1	Organ	MicroCT	36	Compression	Global (Elastix-Transformix)	2000	395–2040 (zero-strain)	Bone failure
Kusins et al. (2020a)	Scapula	Human scapula	3	Organ	MicroCT	33.5	Compression	Global (BoneDVC)	∼1,000	366 (zero-strain)	Bone failure and validation models
Kusins et al. (2020b)	Humeral head	Human humerus	6	Organ	MicroCT	33.5	Compression	Global (BoneDVC)	∼1,000	518 (zero-strain)	Bone failure and validation models
Martelli et al. (2021)	Proximal femur	Human femur	4	Organ	SR-MicroCT	30	Compression	Global (BoneDVC)	1,500 (several reported)	∼1,000 (zero-strain)	Bone failure
Ryan et al. (2020)	Femoral head	Human femur	5	Organ	MicroCT	39	Compression	Global (BoneDVC)	1950 (several reported)	437–612 (zero-strain)	Bone failure
Ridzwan et al. (2018)	Proximal femur	Human femur	14	Organ	QCT	800–1,000	Compression (fall)	Local (Davis)	38,400–48000	300–500 (zero-strain)	Bone failure and validation models
Giorgi and Dall'Ara, (2018)	Tibia	Mouse tibia	3	Organ	MicroCT (*in vivo* protocol)	10.4	Compression	Global (BoneDVC)	520 (several reported)	∼450 (zero-strain)	Bone failure and measurement reproducibility
Madi et al. (2020)	Proximal tibia	Mouse tibia	NA	Organ/Tissue	SR-NanoCT	0.8	Indentation	Local (CPPi)	40	NA	Displacements in calcified cartilage
Liu and Morgan, (2007)	Trabecular bone	Different bone structures	12	Tissue	MicroCT	36	Zero-strain	Local (different parameters)	1,440	∼150–250 (zero-strain)	Precision DVC
Zwahlen et al. (2015)	Trabecular bone	Human vertebra	3	Tissue	SR-MicroCT	7.4	Compression	Global (Demons)	NA	NA	Bone failure
Turunen et al. (2020)	Trabecular bone	Human femur	13	Tissue	SR-MicroCT	3.6	Compression	Local (TomoWarp2)	36	934 (zero-strain)	Bone failure
Yan et al. (2020)	Trabecular bone	Human femur	2	Tissue	SR-MicroCT	3.25	Bending	Local (Davis)	208	NA	Crack propagation in bone
Pena Fernandez et al. (2018a)	Trabecular bone	Ovine femur	4	Tissue	SR-MicroCT	2.6	Compression	Local (Davis)	166.4	510	Effect of radiation on bone properties
Pena Fernandez et al. (2018b)	Trabecular bone	Bovine femur	4	Tissue	SR-NanoCT	0.81	Compression	Local (Davis)	25.9	NA	Effect of radiation and temperature on bone properties
Zauel et al. (2006)	Trabecular bone	Human femur and vertebra	2	Tissue	MicroCT	35	Compression	Local (CCPi)	1,050	168 (zero-strain)	Validation models
Chen et al. (2017)	Trabecular bone	Human and bovine femur	3	Tissue	MicroCT	19.34, 34.44	Compression	Global (BoneDVC)	413–496	NA	Validation models
Knowles et al. (2021)	Trabecular bone	Human humeral head	6	Tissue	MicroCT	5	Compression	Local (Davis)	160	∼550	Validation models
Palanca et al. (2017)	Trabecular bone, cortical bone	Bovine femur, mouse tibia	11	Tissue	SR-MicroCT	1.6	Zero-strain	Global (BoneDVC)	80 (several reported)	∼100–350	Precision DVC
Palanca et al. (2015)	Trabecular and cortical bone	Bovine femur	2	Tissue	MicroCT	9.96	Zero-strain	Global (BoneDVC) Local (Davis)	∼478 (several reported)	Global: 202–243 Local:359–374	Precision DVC
Christen et al. (2012)	Cortical bone	Mouse femur	3	Tissue	SR-NanoCT	0.74	Crack opening	Global (Demons)	18.5	∼20,000 (virtually moved)	Crack propagation in bone
Karali et al. (2021)	Femur	Rat femur	7	Tissue	MicroCT	12	Compression	Local (Davis)	576	<300	Effect of fracture healing on bone mechanics
Karali et al. (2020)	Cortical bone	Bovine femur	12	Tissue	MicroCT	4.2	Indentation	Local (Davis)	201.6	∼220–450	Deformation in indented bone
Jang et al. (2021)	Alveolar socket	Rat mandible	2	Tissue	MicroCT	NA	Compression	Local (Davis)	NA	NA	Deformation of periodontal ligament
Tozzi et al. (2014)	Trabecular bone, cortical bone, bone- cement	Bovine Iliac crest	3	Tissue	MicroCT	20	Compression	Local (Davis)	640	NA	Failure bone/cement interface
Tozzi et al. (2017)	Trabecular bone, cortical bone, trabecular bone with bone cement	Porcine vertebrae with bone cement	5	Tissue	MicroCT	39	Zero-strain	Global (BoneDVC) Local (Davis)	∼1875 (several reported)	Global:∼35–51 Local:∼45–159	Precision DVC
Wearne et al. (2022)	Trabecular bone with or without metal implant	Human tibia	9	Tissue	MicroCT	42	Zero-strain	Local (Davis)	1,180	88–261	Precision DVC
Joffre et al. (2017)	Trabecular bone, metal screw	Lapine femur	4	Tissue	MicroCT	6.5	Screw pull out	Local Forsberg et al. (2008)	208	184 (virtually moved)*	Deformation bone around screw
Pena Fernandez et al. (2022)	Trabecular bone with biomaterial	Ovine distal femur	8	Tissue	MicroCT	5	Compression	Local (Davis)	200	∼200	Validation models
Le Cann et al. (2020)	Implanted tibia	Implanted rat tibia	4	Tissue	SR-MicroCT	25	Screw pull-out	Local (TomoWarp2)	100	NA	Implant stability
Tozzi et al. (2020)	Osteochondral plug	Bovine tibia	4	Tissue	MicroCT	2.02–2.56	Compression	Local (Davis)	∼96	∼200 (zero-strain)	Deformation cartilage
Clark et al. (2020a)	Osteochondral plug (stained PTA)	Human femoral condyles	4	Tissue	MicroCT	1.82–1.87	Compression	Local (Davis)	75	1800 (zero-strain)	Deformation cartilage
Disney et al. (2019)	IVD	Rat IVD		Tissue	SR-MicroCT	1.625	Compression	Local (CCPi)	32.5	NA	Deformation IVD
Lu et al. (2019)	Dentin	Elephant tusk	3	Nano	NanoCT	0.15	Indentation	Local (Davis)	∼3	∼300 (unloaded region)	Dentin fracture mechanics

While these assessments are performed mainly in hard tissues and biomaterials that do not need contrast enhancing in CT images, for soft tissues (e.g., cartilage, IVD) staining techniques have been applied to improve the visualisation of microstructural features and, therefore, improve the assessment of the local deformation using DVC (Section 3.2).

**FIGURE 1:**
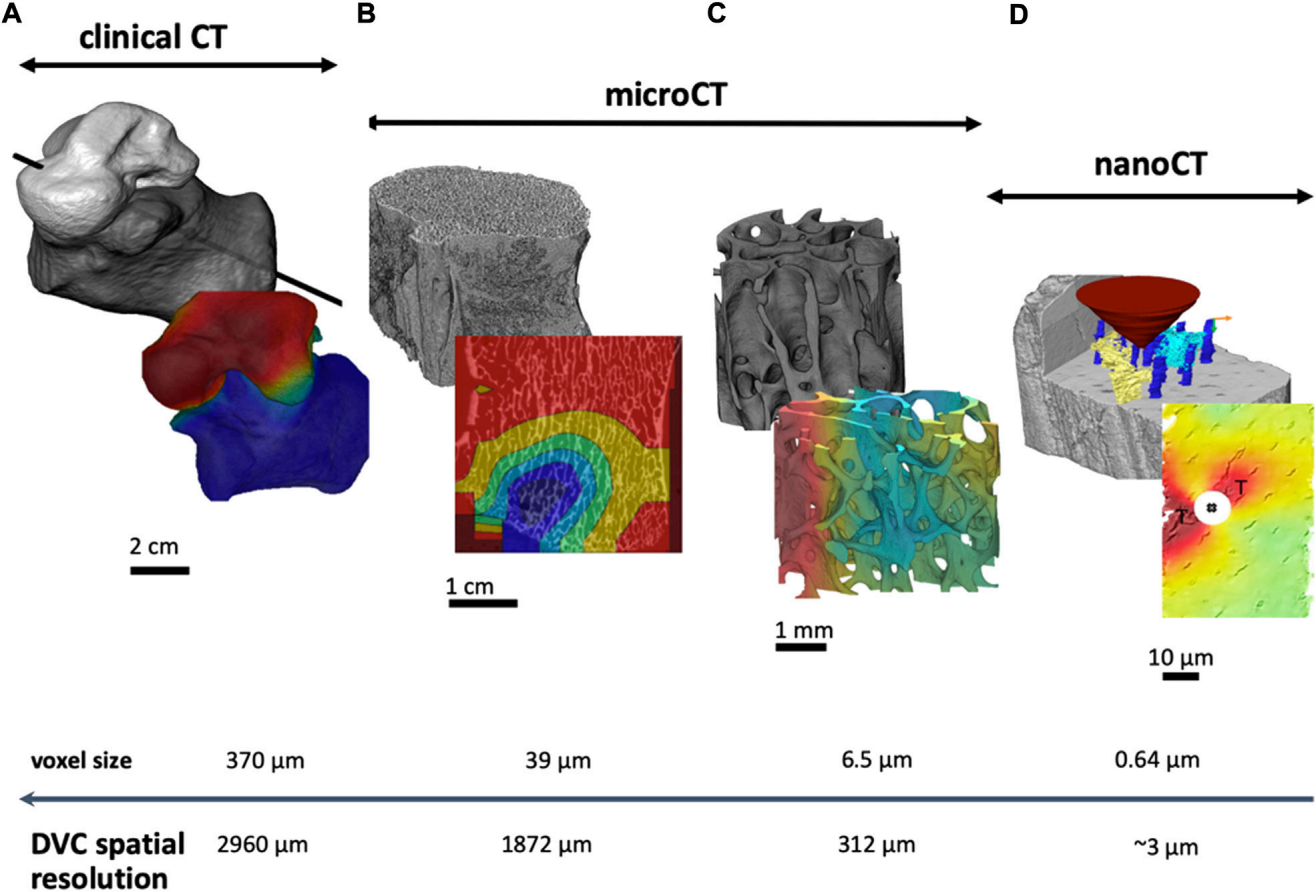
Multi-scale approach based on data acquired from different systems from clinical CT to nanoCT. According to the image source and voxel size, the spatial resolution of the measurement enables coarser or finer DVC evaluation. This is very important when planning an experiment to ensure reliable measurements. References: **(A)** (Pena Fernandez et al., 2020b); **(B)** (Tozzi et al., 2016); **(C)** (Pena Fernandez et al., 2021); **(D)** (Lu et al., 2019).

Depending on the size of the studied specimen (field of view, FOV) and the resolution of the biomedical image used for the DVC analyses, assessments of the deformation at different dimensional scales can be performed, spanning from the organ to the nano level (Figure 1). For example, bone analyses on large portions of the organ can be performed using clinical CT (Ridzwan et al., 2018), on biopsies with microCT (Pena Fernandez et al., 2018a), and on the bulk tissue with SR-microCT (Lu et al., 2019). Nevertheless, it should be noted that we need to accept a compromise between the size of the FOV and the spatial resolution of the DVC approach. This is due to two typical inverse relationships between the size of the FOV and the image resolution, and between the spatial resolution of the DVC and its accuracy (Dall'Ara et al., 2014). However, a recent study on the deformation of the human proximal femur has shown the feasibility of acquiring a large FOV at high resolution with SR-microCT and, therefore, to evaluate the failure mechanism of a large portion of organ with a relatively good DVC spatial resolution (Martelli et al., 2021). Nevertheless, it should be noted that in that case longer scanning time is needed, which is associated with potentially high ionising radiation, which may affect the bone mechanical properties and its deformation (Section 3.4). In that study the DVC approach was applied at the organ level (whole proximal femur) and at the tissue level (femoral head). First DVC applied to the proximal femur using subsampled images (120 μm) to reduce the DVC calculation time. Afterwards, more detailed analyses on the region where the strains localised (femoral head) was performed with the full resolution images (30 μm). This multi-scale application of the DVC approach could be expanded in the future. In fact, some of the current imaging techniques (e.g., Zeiss Xradia Versa X-ray microscopes with Scout-and-Zoom option) enable a multiscale scan of the same specimens, starting from a low-resolution scan of the whole object, and then zooming into interesting regions for high-resolution scans. So far, this approach has been used to highlight differences on the residual strain distribution in cortical bone after cyclic loading at different dimensional scales. By increasing the resolution from 5 µm to 2 µm for the multiscale microCT imaging finer details of the cortical canal network and osteocyte lacunae were revealed. This resulted in improved DVC spatial resolution from 320 µm to 96 µm, which led to different internal strain distributions for the overall scan when compared to the zoom-in region (Pena Fernandez et al., 2020a).

### 2.3 A unique approach to study complex deformation and validate computational models

While standard DVC requires time-lapsed imaging, and therefore slow step-wise loading, it is the only experimental technique that can measure the localization of complex deformations within the heterogeneous tissues. This fundamental feature of DVC enables the comprehensive study of the relationship between the tissue’s microstructure and its mechanical competence (Pena Fernandez et al., 2021), highlighting the effect of local defects or gradients in structure properties (e.g., density, orientation). Nevertheless, it should be noted that the assessment at low dimensional scales can also be done with a combination of high-resolution imaging (e.g., SR-microCT) and highly accurate DVC approaches that allow for a reasonably low sub-volume size.

DVC can also be used to study the deformation associated with the internal material interfaces [e.g., bone and calcified cartilage (Madi et al., 2020), bone and cartilage (Tozzi et al., 2020), bone and biomaterials (Joffre et al., 2017)]. The assessment of the localization of the deformation around the tissue-biomaterial interface can be used to optimise the design of medical devices or to study the integration of biomaterials and native tissue, especially when applied to material collected from animal studies (Pena Fernandez et al., 2019).

While DVC measurements require complex *in situ* mechanical testing and (usually) high resolution imaging, which may not be applicable in most *in vivo* conditions, the experimental assessment of local displacement and strain fields can be used to validate computational models based on laboratory or clinical images. In fact, DVC has been first used to validate the predictions of micro-Finite Element (microFE) models of trabecular bone (biopsy level) generated from microCT images (Zauel et al., 2006; Chen et al., 2017). Further validation has been performed to validate microFE models of more complex structures such as vertebral bodies with (Palanca et al., 2022) or without (Costa et al., 2017) lesions, and the mouse tibia (Oliviero et al., 2018). Recently, more complex models of trabecular bone with heterogeneous material properties (Knowles et al., 2021) or material nonlinearities (Pena Fernandez et al., 2022) have also been validated with DVC data. Finally, the DVC has also been used to validate the outputs of homogenised FE models based on clinical CT images including the human proximal femur (Ridzwan et al., 2018), scapula (Kusins et al., 2020a), humeral head (Kusins et al., 2020b), and vertebrae (Jackman et al., 2016).

## 3 Open challenges and current/future developments

Despite the steep growth of DVC applications in musculoskeletal research and bioengineering over the past 10 years, there are still some aspects that require further attention and research to fully exploit the technique for a variety of tissues, imaging modalities and loading conditions. This section will also suggest some potential avenues to empower/enrich DVC analysis for future work in this area.

### 3.1 Faster measurements and analysis

Conventional *in situ* microCT mechanics is performed in a stepwise manner (time-lapsed testing) where each loading is followed by a “holding period” to perform a full tomographic acquisition, which is less affected by moving artifacts due to stress relaxation. This is particularly important for all biomaterials as they exhibit a certain extent of viscoelasticity increasing from hard to soft tissues. To such extent, DVC analysis based on time-lapsed tests cannot be used to capture the physiological strain mechanisms in biological materials, as their time-dependent response may alter the local measured strains. In response to this need, major efforts have been made to perform fast imaging and time-resolved *in situ* SR-microCT experiments to capture the complex deformation and local strain-damage relationship of bone and biomaterials (Pena Fernandez et al., 2021), which can be further extended to soft tissues and hard-soft interfaces showing higher viscoelasticity and time-dependent mechanical behaviour. A possible route to potentially improve speed and efficiency of DVC is by implementing, for example, Projection-based DVC measurements (P-DVC), which has the advantage of high temporal resolution and a continuous loading (Jailin et al., 2019). The method has been used so far to evaluate engineering materials with fairly resolvable features and would be interesting to see its applicability to complex biological structures. Another way forward to improve efficiency in DVC performance is *via* its integration with machine learning strategies. Deep learning has already proven a powerful tool to classify bone tissue deformation stages to fracture using high-resolution SR-microCT data (Shen et al., 2021). Deep learning-based measurements have been recently introduced as an evolution of 2D DIC (Boukhtache et al., 2021) or for a DVC application (Duan and Huang, 2022), which in both cases was shown to greatly reduce computational complexity of the analysis and therefore improving efficiency. This area has a huge potential to improve measurement and generate more advanced models for understanding and prediction of musculoskeletal tissue mechanics.

### 3.2 Digital volume correlation of soft tissues

The use of DVC to extract local mechanical properties of musculoskeletal soft tissues and hard-soft interfaces is indeed very important and attractive. To this remit a few considerations are needed, particularly in terms of the imaging techniques/modalities used to acquire tissues under loading and consequently the ability of DVC to correlate those image patterns and output displacement/strain. One of the main aspects is in relation to how imaging setup and sample preparation could resolve the texture needed to run DVC. This can be done by employing imaging techniques that are historically developed and refined for soft biomaterials (e.g., confocal microscopy, second/third harmonic generation) where DVC has been used, for example, in strain measurements of the extracellular matrix (Roeder et al., 2004), tendons (Khodabakhshi et al., 2013), tendon cell-induced extracellular matrix deformations (Fung et al., 2018), a variety of other soft materials (Mac Donald and Ravichandran, 2019) and even thin sections of bone (Wentzell et al., 2016). Optical coherence tomography (OCT) combined with DVC is also a promising technique to investigate soft tissues. OCT-based DVC applications include measurement of displacement/strain fields in porcine aortic tissues (Acosta Santamaría et al., 2018) and biomechanical strains in human ocular tissue of patients affected by glaucoma (Czerpak et al., 2022). Its further implementation in musculoskeletal tissues such as articular cartilage and tendon/ligament would surely contribute to advance understanding of their biomechanics. Another image modality that was recently explored in combination to DVC is magnetic resonance imaging (MRI). MRI-based DVC application has been successfully used to obtain the full-field strain in human intervertebral disc (IVD) *ex vivo* (Tavana et al., 2020b). However, due to the limitations in volume imaged with confocal or availability/cost/resolution of MRI, the main input image for DVC is still from X-ray CT which is inherently less suitable to resolve soft tissues. To overcome this problem two main solutions are typically used to enhance structural features for DVC by either employing in-line phase-contrast imaging (achievable in SR-microCT like setups) or staining of the tissues. Interesting applications of DVC in soft tissues from phase-contrast SR-microCT images include the visualisation of microstructural deformation (Disney et al., 2019) and localised measurement of fibre-level orientation, curvature and strain of native intact intervertebral disc (Disney et al., 2022). Phase-contrast has been also exploited in laboratory microCT to improve contrast in native articular cartilage and mineralized tissue to quantify residual strain distribution at the cartilage-bone interface (Tozzi et al., 2020). Unfortunately, phase-contrast *in situ* mechanics in laboratory systems expose tissue to long periods of irradiation, which could affect its integrity and consequent measurements as will be further discussed in Section 3.5. Another route to enhance CT contrast in soft tissues is *via* radiopaque staining (e.g., iodine potassium iodide, phosphotungstic acid). A recent study relied on previously developed staining protocols (Clark et al., 2020b) to resolve chondrocyte distribution in human articular cartilage and measure full-field displacement and strain using DVC (Clark et al., 2021), but once again, most staining procedures can alter both tissue morphology and mechanical properties (Buytaert et al., 2014). New generation of staining agents with the potential of better preserving tissue integrity (de Bournonville et al., 2020) could address the degradation problem; however, even in such case, issues related to heterogeneity of tissue contrast due to staining penetration/power can notably reduce DVC ability to measure reliable and reproducible local deformations.

### 3.3 Increase the digital volume correlation accuracy/precision and validation

The accuracy and precision of DVC approaches have been comprehensively assessed using repeated microCT scans (Dall׳Ara et al., 2017; Liu and Morgan, 2007) or virtually deformed repeated microCT scans (Comini et al., 2019; Ryan et al., 2020), which consider the input image noise in contrast with the virtually moved or deformed images. While these assessments are required to provide a level of confidence of the DVC measurements, which are extremely dependent from the signal-to-noise ratio in the input images, the heterogeneity of the tested material, and the DVC parameters, these approaches are based on homogeneous (usually zero-strain fields) or simple (usually affine deformations) strain fields, which are unrealistic for most biological applications. Therefore, future tests of accuracy based on realistic heterogeneous deformations (e.g., from finite element model simulations) would provide a better assessment of local DVC errors.

From the standard assessment of DVC uncertainties it has been demonstrated for different bone structures that the precision increases by decreasing the spatial resolution of the DVC and that the precision depends on the image quality of the input images and in particular on how many features are clearly identified in them. Combining these properties it becomes clear that in order to increase the spatial resolution of the DVC input images with higher resolution and signal-to-noise ratio are required: for example the spatial resolution of the DVC increases of 13 times when applied to SR-microCT images (voxel size 1.6 μm) of the mouse tibia compared to *in vivo* microCT images (resolution 10.4 μm) of the same object. Nevertheless, while high-resolution images can be obtained using high-resolution laboratory microCT scanners or SR-microCT facilities, it should be noted that these imaging modalities are likely to perturb the mechanical competence of the scanned biological tissue (see Section 3.4).

### 3.4 Reducing invasiveness of X-ray imaging

It is established that prolonged X-ray exposure may severely alter the structural integrity and mechanical properties of musculoskeletal tissues, particularly in high-flux setups such as SR-microCT as extensively reported in a recent study (Dall'Ara et al., 2022). Briefly, as for SR-irradiated bone the degradation of the tissue and its mechanical properties is due to the alteration of collagen cross-linking (Barth et al., 2010; Barth et al., 2011), in soft tissues like articular cartilage degradation of mechanical properties has been instead associated with the decline in proteoglycan synthesis (Cicek, 2016). DVC has played a vital contribution in this area by further evaluating the effect of SR-microCT radiation on trabecular bone (Pena Fernandez et al., 2018a) and proposing strategies to mitigate this issue by reducing temperature in the *in situ* experiments (Pena Fernandez et al., 2018b). Despite such great efforts, more research is needed to clearly identify the mechanism of radiation-induced tissue degeneration and achieve the best compromise between image quality, number of *in situ* steps during the experiment and potential damage. All this is inevitably reflecting on the DVC analysis from such images and it is important to properly plan future studies where this potential issue is carefully evaluated, contextualising the DVC-based measurements with at least proper estimations of the radiation exposure on the specimens, to avoid reporting DVC results which may be affected by the invasiveness of the imaging modality.

### 3.5 Integrating mechanics in digital volume correlation inputs

The DVC algorithm usually ignores the local mechanical properties of the studied objects. In fact, the image correlation is completely unaware of the local material properties of the imaged object, but can only take into account for the features that affect the local porosity or anisotropy, if visible in the acquired input images. Nevertheless, the deformation of the considered tissue is also a function of those material properties that do not directly affect the image taken at a certain dimensional scale. For example, the material properties of the extracellular matrix of bone may be different from specimen to specimen (e.g., healthy tissue vs. tissue from osteogenesis imperfecta patients, which are more fragile for a molecular mutation in the collagen), even in specimens with similar bone mineral density and microstructure. While some DVC algorithms have been integrated with finite element solvers to consider the mechanical heterogeneity of the studied structures and back calculate their constitutive behaviour [i.e., the parameters of the chosen constitutive law), so far this approach has been used only for engineered materials (e.g., metamaterials (Valmalle et al., 2022)]. Applications to biological tissues with hierarchical organisation of the structure and the local heterogeneity in material properties due to local differences in mineralization (e.g., bone) or organisation of fibres (e.g., tendons, intervertebral disc), would require fitting complex constitutive laws and require further development.

Moreover, standard DVC analysis can only provide information about displacement and strain fields, with no information about the internal loads and stresses in the microstructure. DVC has been extensively used in the last decade to validate the displacement and strain outputs of FE models built from the undeformed images, both at the microstructural (microFE) (Palanca et al., 2022) and continuum (Kusins et al., 2020a) levels for bone. The validated FE models have then the potential to evaluate stresses in the musculoskeletal tissue, using the DVC data only to assign the proper boundary conditions to the FE models. Nevertheless, this approach has not been applied yet to study the local stresses in musculoskeletal tissues.

### 3.6 Clinical applications of digital volume correlation

A very important implementation of DVC is in the mechanical interpretation of clinical imaging. The technique was used to quantify the 3D full-field displacement and measure the helical axis of the subtalar joint *in vivo* during inversion-eversion in full weight-bearing clinical CT, providing a better understanding of the relative motion at the subtalar joint under physiological loading (Pena Fernandez et al., 2020b). MRI-based DVC was also used to assess *in vivo* strain uncertainties in the human talus, showing its potential to measure relevant levels of *in vivo* bone strain and to be used for a range of clinical applications (Tavana et al., 2020c). Another interesting application is the digital tomosynthesis (DTS) based DVC, which was employed to calculate full-field vertebral displacement maps, and in turn stiffness, using *in vivo* images acquired in both standing and standing-with-weight (8 kg) configurations (Oravec et al., 2019). Altogether, such use of DVC is very promising and can provide valuable insights in clinical diagnostics and surgical planning.

## 4 Conclusion

The goal of this minireview was to highlight the strengths, limitations and potential of the DVC approach to study the deformation of musculoskeletal tissues. Considering the limited space, the comprehensive review of every DVC study on musculoskeletal tissues was not in the remit of this manuscript. The authors hope that the reader will find this review useful and stimulating to engage with the usage of DVC for musculoskeletal research, to tackle current challenges and to further develop this approach in the musculoskeletal field and beyond.
